# A Study of HDV in HBsAg Positive Patients in Tabriz, Northwestern Iran

**Published:** 2010-06-01

**Authors:** Sirus Jedary Seifi, Masoud Sabouri Ghannad

**Affiliations:** 1 Department of Microbiology, Faculty of Medicine, Tabriz University of Medical Sciences, Tabriz, Iran; 2 Department of Microbiology, Faculty of Medicine, Hamadan University of Medical Sciences, Hamadan, Iran

**Keywords:** HDV, Haemophilia, Hemodialysis, Thalassemia, Intravenous Drug Users

## Abstract

**Backgrounds and Aims:**

Hepatitis Delta Virus (HDV) is a defective ssRNA virus requiring the provision of hepatitis B virus (HBV) for packaging of new HDV virions. Since the epidemiological features of HDV in this part of Iran seem to be unknown, the aim of this research was to determine the seroprevalence of HDV of hepatitis B surface antigen positive (HBsAg+) in blood donors, injecting drug users, hemophiliacs, hemodialysis and thalassemic patients in the city of Tabriz in northwestern Iran.

**Methods:**

The numbers of patients who were screened in the years 2006-2007 were 100 hemodialysis patients, 165 blood donors and 90 intravenous drug users, or a total of 355 patients who were HBsAg+ and randomly selected at the Tabriz Regional Educational Blood Center. Anti-HDV antibodies (IgM), alanine aminotransferase (ALT) and aspartate aminotransferase (AST) levels in serum samples were measured.

**Results:**

Eight of the hemodialysis patients (8%), 3 of the 165 blood donors (1.8%) and 9 intravenous drug addicts (10%) were HDV-IgM antibody-positive. Out of 152 serum samples collected in the hemophiliac population, only 11 samples were HBsAg+ (7.23%), and anti-HDV IgM antibodies were detected in just 2 cases. Also out of 112 thalassemic patients’ sera specimens, only 2 samples was HBsAg+, and HDV-IgM antibodies were negative. The seropositivity of anti- HDV antibodies was 6.01%. The study of serum ALT levels in HBV-positive and HDV-positive patients showed obvious elevation in more than 95% of cases.

**Conclusions:**

The results show the endemicity of HDV infection in Tabriz. HDV infection in Iran could be controlled by nationwide HBV vaccination.

## Introduction

Hepatitis B is one of the most common infectious diseases, with about 350 million infected people worldwide, predominantly (75% to 80%) in Asia and Eastern Europe [1]. About 1 million deaths annually are estimated to be caused by hepatitis B infection, which has been the main risk factor for cirrhosis and hepatocellular carcinoma worldwide [2]. More than 3% of Iranians has been reported to be hepatitis B virus (HBV) positive [3][4]. Hepatitis Delta virus (HDV), is a defective ssRNA virus, in which HBV surface proteins are needed for packaging of new HDV virions: it is usually studied in hepatitis B surface antigen-positive (HBsAg+) patients [5][6][7][8]. Acute and chronic liver diseases have been reported in patients infected with HDV. It is estimated that more than 15 million patients are infected with HDV. The incidence of HDV infection in the western regions of Asia, Eastern Europe and Italy is high in comparison with the rest of the world [9][10][11]. HDV has been reported to be endemic in the Middle East [12]. A considerable body of evidence now suggests that simultaneous infection with HDV leads to an acceleration of the progress of chronic HBV infection into chronic hepatitis, cirrhosis and finally, hepatocellular carcinoma [13]. It has also been reported that co-infection or super-infection with HDV may occur in 25% of chronic HBV carriers. Only 15% of infected patients with HBV develop cirrhosis in comparison to 70%–80% of HDV-infected patients who develop cirrhosis [14].

Since the epidemiological features of HDV in northwestern Iran seem to be unknown, the aim of this research was to manage the epidemiological features of HDV infection in high-risk populations in the city of Tabriz, the capital of the province of Western Azarbaijan, one of the 30 provinces of Iran. This study was in line with the objective of investigating the risk of HDV transmission in at-risk populations in the category of HBsAg+, including blood donors, injecting drug users, hemophiliacs, hemodialysis and thalassemic patients in this part of Iran   The reason for using anti-HDV IgM antibodies to make a diagnosis of HDV infection was to ensure that patients had been newly-infected, and the results were due to recent, and not long-term infection. This will facilitate the management of the disease in Tabriz, and the detailed evaluation of the distribution and characteristics of HDV in this part of Iran.

## Materials and Methods

Five different groups of subjects, including hemodialysis patients, blood donors, intravenous drug users, hemophiliac and thalassemic patients were studied. The tests were performed as part of a routine clinical assessment. Out of the 619 patients who were studied in this research, 100 patients were hemodialysis patients, 165 were blood donors and 90 were intravenous drug users. In total, 355 patients who were HBsAg+, were randomly selected at the Regional and Educational Division of the Blood Transfusion Organization Research Center. Their serum samples were screened for immunoglobulin M antibodies to hepatitis delta virus (anti-HDV IgM), using Diapro kits and the enzyme-linked immunosorbent assay (ELISA) method. Out of 152 hemophiliac and 112 thalassemic patients, only 11 and 2 samples, respectively, were HBsAg+. ALT and AST levels in serum samples were measured by the IFCC method using Pars Azmoon kits. The Fisher exact and chi-square tests were performed to analyze the data obtained by using SPSS, version 13.

## Results

Among the 619 patients screened in the years 2006-2007. 100 hemodialysis patients, 165 blood donors and 90 intravenous drug users, who were HBsAg+, were screened for anti-HDV IgM. Eight of the hemodialysis patients (8%), 3 of the 165 blood donors (1.8%) and 9 of the intravenous drug users (10%) were anti-HDV IgM positive. Out of 152 serum samples collected from hemophiliac patients, only 11 samples were HBsAg+ (7.23%), and anti-HDV IgM antibodies were detected in just 2 (0.18 %) cases in the HBsAg+ patients. Also, out of 112 sera from thalassemics, only 2 samples were HBsAg+, and anti-HDV IgM antibodies were negative in all of the patients (Table 1). A study of serum ALT levels in HBV+ and HDV+ patients showed obvious elevation (100%) in all of them (Table 2).

**Table 1:** Distribution of the blood donors, injecting drug users, hemophiliacs, hemodialysis and thalassemic patients studied in this research according to residential area, occupation, the level of literacy and marital status in Tabriz.

**FactorsUnderStudy**	**Residential Area**		**Occupation**		**Marital status**	
**Groups Under Study**	**Tabriz**	**County**	**Govermental**	**NGO**	**Single**	**Married**
**Blood Donors**	58.78%	41.22%	36.36%	63.64%	38.79%	61.21%
**Hemodialysis Patients**	72%	28%	44%	56%	68%	32%
**Hemophiliacs**	55.26%	44.74%	32.24%	67.76%	25.66%	74.34%
**Injecting Drug Users**	83.33%	16.67%	18.89%	81.11%	30%	70%
**Thalassemics**	80.36%	19.64%	-	-	100%	-

**Table 2:** The comparison of level of liver enzymes (AST and ALT) in HDV+ and HDV- patients studied in this research.

**Groups Under Study**	**ALT**		**AST**	
	**(HDV+)**	**(HDV-)**	**(HDV+)**	**(HDV-)**
**Blood Donors**	n=3 100%>normal	n=162 50%> normal	n=3 100%>normal	n=162 45%> normal
**Hemodialysis Patients**	n=8 100%>normal	n=92 60%> normal	n=8 100%>normal	n=92 60%> normal
**Injecting Drug Users**	n=9 100%>normal	n=81 60%> normal	n=9 100%>normal	n=81 65%> normal
**Hemophiliacs**	n=2 100%>normal	n=9 60%> normal	n=2 100%>normal	n=9 60%> normal
**Thalassemics**	- normal	n=112 normal	-normal	n=112 normal

## Discussion

The present study is, to the best of our knowledge, one of the few published papers which has organized the epidemiological data about the co-infection of HDV among HBsAg+ simultaneously into the categories of blood donors, injecting drug users, hemophiliacs, hemodialysis and thalassemic patients in Tabriz’s population. We conducted an epidemiological study of 619 patients, and based on the data obtained, we have understood that the seropositivity of anti-HDV antibodies was 6.01%, showing the endemicity of HDV infection in Tabriz, consistent with the results (6%) that have been previously reported among HBsAg+ subjects from Tabriz [15]. This shows that the seroprevalence of HDV antibodies has been almost constant over a period of about 5 years, in this part of Iran. This is in contrast with another report from Golestan, a northeastern province in Iran, which shows an increasing rate of HDV infection during the last decade [16]. The results obtained in this research were significantly higher than in the reports  in some previous Iranian research[17][18]. HDV antibodies were reported in 2.5% of the asymptomatic HBsAg+ population [17]. In a similar study from Hamadan, a western province of Iran, a similar prevalence (2.4%) of HDV infection was reported [18]. Hassanjani-Roshan and Taheri, in 2000, reported an HDV seroprevalence of 2% in HBsAg+ carriers in Babol, another city in northern Iran [4], which is a finding consistent with previous reports from Iran. Another report indicates a HDV seroprevalence of 5.7% in Iran, among HBV-infected patients [19]. The prevalence of HDV infection among the HBsAg+ population has been reported to be 24.4%, 16.6%, 4%, 2.2%, 1.6%, and 1.5%, in Bangladesh [20], Pakistan [21], Mexico [22], Taiwan [23], Spain [24], and Yugoslavia {%25), respectively.

From Table 1, it can be concluded that in terms of patients’ place of residence, whether in rural or in urban areas, the majority of the population under study were living in an urban area, in Tabriz, the capital of the province of Western Azarbaijan, as opposed to a rural area. In terms of occupational status, 63.62%, 56%, 67.76% and 81.11% of the patients were employed by non-governmental organizations (NGO) and were members of the following groups, respectively: blood donors, hemodialysis patients, hemophiliacs and injecting drug users.

As can be seen in Table 3, 72.72%, 69%, 61.84%, 77.77% and 53.57% of the patients under study were blood donors, hemodialysis patients, hemophiliac patients, thalassemic patient and injecting drug users, respectively. Table 3 also shows that antibodies against HDV were positive in 22 patients, including 20 males and 2 females. Consequently, this study shows that among infected patients, males (90%) were more often infected with HDV than were females (10%). This might be due to high-risk behavior in men, such as intravenous drug abuse. These results are consistent with previous reports from Babol, another city in northern Iran [4] and from Pakistan [21]. One of the limitations that we faced in this study was the  number of males and females under study, which was unequal, a factor which might have affected the results obtained in this research. On the other hand, the data obtained in this study has suggested that gender may be connected with HDV infection. However, we did not find any significant relationship between HDV infection and residential area, occupation and marital factors.

Based on the data shown in Table 4, it can be concluded that 3 of the blood donors (1.81%), 8 of the patients undergoing hemodialysis (8%), and 9 of the intravenous drug users (10%), had anti-HDV IgM antibodies. Out of 152 serum samples collected from hemophiliacs, HDV-IgM antibodies were detected in just 2 cases, (1.3%) which is a lesser number than in reports from some other countries. Troisi et al., 1993 showed 9.1% of anti-HDV in the hemophiliac population of the United States. Also, in all 112 patients with thalassemia, antibodies against HDV-IgM were negative.

A research study in India showed that 16.7% of HBsAg+ multi-transfused thalassemics had antibodies against HDV [26]. A similar study performed on thalassemic children in Bangladesh showed no positive antibodies [27], the latter results being consistent with our current findings. From Table 4, it can also be concluded that the higher rates of anti-HDV IgM antibodies were seen in single people living in Tabriz and among NGO personnel. This would indicate the possibility of risk-taking behaviors among these groups.

Table 2 shows an obvious elevation in the level of liver enzymes in the serum of all patients, including ALT and AST levels, in HBV+ and HDV+ patients.

Huo et al., 2004 showed that 5% of injecting drug users studied in Taiwan had HDV antibodies [28]. A study performed by Cross et al., 2008, on injecting drug users showed that 28.1% of the population under study in Eastern Europe, 26.8% in Africa and 7.3% in the Middle East had antibodies against HDV [29]. Chen et al., 1992 showed a 2.2% positive anti-HDV in blood donors in Taiwan [23] in comparison to the rate in Indonesia   .5%) [30]. Torabi et al., 2002, showed that 4.6% of HBsAg+ blood donors in Tabriz [15] had antibodies against HDV, whereas our study showed 1.81% anti-HDV in this population. This shows a declining rate of 2.59% in this group of people over a period of 5 years, which may show the effects of vaccination in this region of Iran. On the other hand, since injecting drug users were listed as the largest group of HDV-infected patients in Tabriz, our research suggests that HDV screening is essential among injecting drug users. Continuous diagnostic laboratory testing may show the way to the most rational therapeutic strategy in overcoming the disease.

Some countries have organized successful sanitation projects to insure the decline of the prevalence of HDV infection [11][31][32][33]. Our results suggest that in Tabriz, HDV infection, as well, can be controlled. Since 2005, a national plan has been developed in Iran to vaccinate teenagers against HBV. Efforts to reduce HBV and consequently, HDV, in Tabriz and in other provinces can be implemented by means of broad-scale vaccination in the country. However, essential research by practitioners and health-care managers alike is required for the evaluation of the effectiveness of HBV vaccination programs in controlling HBV, and consequently HDV. This effort could provide valuable information on the circulation of HDV. The Iranian authorities could prioritize government programs to control HDV infection nationwide. A regulatory HDV committee and subcommittees could be established as HIV committees and subcommittees have been, which have so far been known to be very effective. Also in each province of Iran, representatives of blood transfusion organizations, the ministry of education, universities of medical sciences, and prison authorities need to perform further research to consider other methods of HDV transmission and to reduce the prevalence of HDV, as future goals.

**Table 3:** Distribution of the blood donors, injecting drug users, hemophiliacs, hemodialysis and thalassemic patients studied in this research according to sex in Tabriz.

**Factors Under Study**	**Sex**		**No. of Positive HDV Antibody**	
**Groups Under Study**	**Male**	**Female**	**Male**	**Female**
**Blood donors**	72.73%	27.27%	2	-
**Hemodialysis Patients**	69%	31%	7	1
**Hemophiliacs**	61.84%	38.16%	2	-
**Injecting Drug Users**	53.57%	46.43%	8	1
**Thalassemics**	80.36%	19.64%	-	-
**P-value**	< 0.004		-	

**Table 4:** Distribution of HDV positive patients studied in this search according to marital status, residential area and occupation in Tabriz

**Factors Under Study**	**Residential Area The Whole No. (HDV+)**		**Occupation The Whole No. (HDV+)**		**Marital Status The Whole No. (HDV+)**	
**Groups Under Study**	**Tabriz**	**Count**	**Governmental**	**NGO**	**Single**	**Married**
**Blood Donors (165)**	97(2)	68(1)	60(1)	105(2)	64(3)	101(-)
**Hemodialysis Patients (100)**	72(4)	28(4)	44(2)	56(6)	68(5)	32(3)
**Hemophiliacs (152)**	84(2)	68(-)	49(1)	103(1)	39(-)	113(2)
**Injecting Drug Users (90)**	75(8)	15(1)	17(1)	73(8)	27(5)	63(4)
**Thalassemics (112)**	90(-)	22(-)	-	-	-	-
**P-value**	0.1		0.8		0.6	
